# Additive Antinociceptive Effects of a Combination of Vitamin C and Vitamin E after Peripheral Nerve Injury

**DOI:** 10.1371/journal.pone.0029240

**Published:** 2011-12-14

**Authors:** Ruirui Lu, Wiebke Kallenborn-Gerhardt, Gerd Geisslinger, Achim Schmidtko

**Affiliations:** pharmazentrum frankfurt/ZAFES, Institut für Klinische Pharmakologie, Klinikum der Goethe-Universität, Frankfurt am Main, Germany; Universidad Federal de Santa Catarina, Brazil

## Abstract

Accumulating evidence indicates that increased generation of reactive oxygen species (ROS) contributes to the development of exaggerated pain hypersensitivity during persistent pain. In the present study, we investigated the antinociceptive efficacy of the antioxidants vitamin C and vitamin E in mouse models of inflammatory and neuropathic pain. We show that systemic administration of a combination of vitamins C and E inhibited the early behavioral responses to formalin injection and the neuropathic pain behavior after peripheral nerve injury, but not the inflammatory pain behavior induced by Complete Freund's Adjuvant. In contrast, vitamin C or vitamin E given alone failed to affect the nociceptive behavior in all tested models. The attenuated neuropathic pain behavior induced by the vitamin C and E combination was paralleled by a reduced p38 phosphorylation in the spinal cord and in dorsal root ganglia, and was also observed after intrathecal injection of the vitamins. Moreover, the vitamin C and E combination ameliorated the allodynia induced by an intrathecally delivered ROS donor. Our results suggest that administration of vitamins C and E in combination may exert synergistic antinociceptive effects, and further indicate that ROS essentially contribute to nociceptive processing in special pain states.

## Introduction

Reactive oxygen species (ROS), such as superoxide, hydrogen peroxide and hydroxyl radicals, are derived from the metabolism of molecular oxygen [1], [2]. Due to their highly reactive nature, ROS can damage nucleic acids, proteins and lipids, especially at high concentrations. Accordingly, excessive production of ROS in the central nervous system (CNS) contributes to the pathogenesis of many neurodegenerative diseases including Alzheimer's disease, Parkinson's disease and amyotrophic lateral sclerosis [3]. Recent studies suggest that ROS at physiological concentrations can mediate reversible regulatory processes and serve as functional messenger molecules, possibly fulfilling a large range of physiologic and pathophysiologic functions [4]. Notably, accumulating evidence indicates that ROS are involved in the sensitization of pain pathways during persistent pain. For example, an increased ROS production has been detected in the spinal cord and in peripheral tissues after noxious hindpaw stimulation or nerve injury [5]–[7]. Mice lacking the superoxide-generating NADPH oxidases Nox1 or Nox2 demonstrated a reduced nociceptive behavior in animal models of inflammatory or neuropathic pain, respectively, indicating a contribution of NADPH oxidase-mediated superoxide production to pain sensitization [8], [9]. Moreover, inflammatory and neuropathic pain was effectively inhibited in animal models after administration of free radical scavengers or superoxide dismutase mimetics [10]–[14], further suggesting that ROS contribute to nociceptive processing.

Vitamin C (Vit C) and vitamin E (Vit E) are essential nutrients that function as antioxidants in the human body. Vit C (L-ascorbic acid) is a water-soluble sugar acid that exists at physiological pH as a monovalent anion, ascorbate, which is capable to scavenge ROS [15]. Vit E (alpha-tocopherol), a fat-soluble vitamin, is the major chain-breaking antioxidant in body tissues and is the first line of defense against lipid peroxidation, protecting cell membranes from free radical attack [16]. Notably, Vit C is able to recycle Vit E by reduction of the tocopheroxyl radical of Vit E, thereby permitting Vit E to function again as a free radical chain-breaking antioxidant [17]. Based on the observation that (i) ROS contribute to nociceptive signaling and that (ii) the natural compounds Vit C and Vit E may exert additive antioxidant effects, we hypothesized that a combination of both vitamins might attenuate persistent pain. Hence, the goal of this study was to evaluate whether or not exogenous Vit C and Vit E are able to inhibit nociceptive behavior in animal models of inflammatory or neuropathic pain.

## Methods

### Ethics Statement

All experiments were approved by the federal authority for animal research (Regierungspräsidium Darmstadt, Hessen, Germany) and were carried out in strict accordance with the National Institutes of Health's Guide for the Care and Use of Laboratory Animals. All efforts were made to minimize suffering.

### Animals

Male C57BL/6 mice (8–9 weeks old, 25±1 g) were obtained from Harlan Laboratories. Animals were maintained under a 12/12-h light/dark cycle with free access to water and standard chow (ssniff® R/M-H; ssniff, Soest, Germany).

### Drugs

Commercially available liquid parenteral formulations of Vit C (Pascorbin®, Pascoe, Giessen, Germany and Vitamin C-Rotexmedica®, Rotexmedica, Trittau, Germany; containing 150 and 100 mg/ml L-ascorbic acid, respectively) and Vit E (Vitamin E Sanum®, Sanum-Kehlbeck, Hoya, Germany and Ephynal®, Bayer, Barcelona, Spain; containing 75 and 50 mg/ml alpha-tocopherol acetate, respectively), or 0.9% saline (B. Braun, Melsungen, Germany) were used. For multiple daily intraperitoneal (i.p.) injections and oral administrations of low vitamin doses, the vitamin formulations were diluted with 0.9% saline. For intrathecal (i.t.) injections (injection volume 2.5 µl), L-ascorbic acid (Sigma-Aldrich, Munich, Germany) was added to the Vit C formulation (Pascorbin®) to obtain a final concentration of 300 mg/ml and NaHCO_3_ was used to adjust pH to 7.0, whereas alpha-tocopherol acetate (Sigma-Aldrich) was added to the Vit E formulation (Vitamin E Sanum®) to obtain a final concentration of 150 mg/ml. Vitamins C and E in combination were administered by consecutive single doses of Vit C and Vit E, respectively. All drug preparations were performed immediately before administration to minimize air oxidation of the vitamin solutions.

### Behavioral Testing

Prior to behavioral testing, animals were habituated to the experimental room for at least 1 h. Experiments were performed by an observer blinded for the treatment of the animals.

#### Formalin test

Twenty or two minutes after i.p. drug injection, 15 µl of a 5% formaldehyde solution (formalin) was injected subcutaneously into the dorsal surface of a hindpaw [18], [19]. The time spent licking the formalin-injected paw was recorded in 5 min intervals up to 60 min after formalin injection.

#### Dynamic-plantar test

The mechanical sensitivity of the plantar side of a hindpaw was assessed with an automated von Frey-type testing device (Dynamic Plantar Aesthesiometer, Ugo Basile) which allows for reliable detection of mechanical sensitivity in mice [20]–[23]. The stainless steel probe of the touch stimulator unit was pushed against the paw with ascending force until a strong and immediate withdrawal occurred. The maximum force was set at 5 g and the ramp speed was 0.5 g/s. The paw withdrawal latency was calculated as the mean of five to six consecutive trials with at least 20 s in between.

#### CFA-induced mechanical hyperalgesia

Twenty microliter of complete Freund's adjuvant (CFA, containing 1 mg/ml of heat killed *Mycobacterium tuberculosis* in paraffin oil 85% and mannide monooleate 15%, Sigma-Aldrich) were injected into the plantar subcutaneous space of a hindpaw [24]. Twenty-four hours after CFA injection, hindpaw withdrawal latencies were determined using the dynamic plantar test to confirm CFA-induced mechanical hyperalgesia. Thereafter drugs were i.p. injected, and hindpaw withdrawal latencies were determined at 1, 2, 4, 6, 24 h after drug injection. Immediately after the 24 h measurement (i.e., 48 h after CFA), animals received a second i.p. injection of the same drug, and hindpaw withdrawal latencies were again determined at 1, 2, 4, 6, 24 h thereafter. For investigation of long-term effects with multiple daily injections, drugs were i.p. administered once a day starting 30 min after CFA injection, and withdrawal latencies were investigated 1 h after drug injections at 1, 3, 5, 7, 10 and 12 d after CFA injection.

#### SNI-induced neuropathic pain

The ‘spared nerve injury’ (SNI) model was used to investigate neuropathic pain. Under general anesthesia (1.5% isoflurane), the tibial and common peroneal branches of the sciatic nerve were ligated and sectioned distally, while the sural nerve was left intact [25]. Mechanical allodynia was determined using the dynamic-plantar test. For investigation of immediate effects of drug injections, drugs were i.p. or intrathecally (i.t.) administered 15 days after SNI surgery and paw withdrawal latencies were determined at 1, 2, 4, 6, 24 h after drug injection. Immediately after the 24 h measurement, drugs were again administered and withdrawal latencies were determined at 1, 2, 4, 6, 24 h thereafter. For investigation of long-term effects with multiple daily dosing, drugs were administered once a day starting 30 min after SNI surgery, and withdrawal latencies were investigated at 3, 6, 8, 10 and 12 d after SNI surgery. Measurements were performed 1 h after i.p. administration, or 1 and 2 h after oral administration (by gavage). For the latter, the mean of both measurements was calculated.

#### Intrathecal injections

For i.t. delivery, drugs were injected by direct lumbar puncture in awake, conscious mice as described [26]. Briefly, a 10 µl Hamilton syringe connected to a 30 gauge, 0.5 inch needle was inserted in a 70–80° angle at the midline between the hip bones (held by the thumb and forefinger of the experimenter). After contact with the bone of the spinal column was sensed, the needle angle was reduced to approximately 30° and slipped in between the vertebrae. Drugs were i.t. injected in a volume of 2.5 µl immediately after a reflexive tail flick or `S ` shape indicating puncture of the dura mater. An accuracy >95% was achieved by dye injections in training sessions prior to the experiment.

#### TBHP-induced allodynia

One hour after i.p. drug injection, 100 µg TBHP (tert-butyl hydroperoxide; Sigma Aldrich) dissolved in 5 µl saline was i.t. injected as described above. Mechanical allodynia was determined using the dynamic-plantar test.

### Western Blot Analysis

Mice were i.p. injected with Vit C and Vit E or 0.9% saline 14 and 15 days after SNI surgery. Three hours after the second drug injection, mice were killed by carbon dioxide, the lumbar spinal cord and dorsal root ganglia (DRGs, L3–L5) were rapidly dissected, immediately frozen in liquid nitrogen and stored at −80°C until use. Tissue samples were homogenized in Phosphosafe extraction reagent (Novagen, Madison, WI) mixed with a protease inhibitor cocktail (Complete Mini; Roche Diagnostics, Mannheim, Germany), and centrifuged at 14,000× *g* for 1 h. Extracted proteins (20 µg per lane) were separated by SDS-polyacrylamide gel electrophoresis and transferred onto nitrocellulose membranes by electroblotting. After incubating in blocking buffer (Odyssey blocking buffer; LI-COR Biosciences, Bad Homburg, Germany; diluted 1∶1 with PBS) for 1 h, membranes were incubated overnight at 4°C with antibodies directed against phopho-p38 mitogen-activated protein kinase (MAPK) (Thr180/Tyr182; 1∶100; Cell Signaling, Danvers, MA), p38 MAPK (1∶100; Cell Signaling), phospho-p44/42 MAPK (Thr202/Tyr204; 1∶100; Cell Signaling), p44/42 MAPK (1∶500; Promega, Madison, WI) or calnexin (1∶100; Santa Cruz Biotechnology, Santa Cruz, CA) diluted in blocking buffer containing 0.2% Tween. After incubation with the secondary antibodies conjugated with Alexa Fluor 680 or 800 (1∶10000; Invitrogen, Carlsbad, CA) for 2 h, blots were visualized on a Odyssey Infrared Imaging System (LI-COR Biosciences). Band densities were quantified by densitometry using the ImageJ 1.43 software (NIH, USA). Data are given in relation to the individual calnexin loading control and are normalized to band intensities of spinal cord extracts of saline-treated animals.

### Statistics

Statistical evaluation was done with SPSS 17.0 for Windows (SPSS, Chicago, IL). The Kolmogorov-Smirnov test was used to assess normal distribution of data within groups. Data were analyzed with one-way or repeated-measures ANOVA followed by Fisher *post hoc* test. For all tests, a probability value *P*<0.05 was considered as statistically significant. All data are expressed as the mean ± SEM, and the error bars represent SEM.

## Results

### A combination of vitamins C and E inhibits the first phase of formalin-induced pain behavior

**Figure 1:**
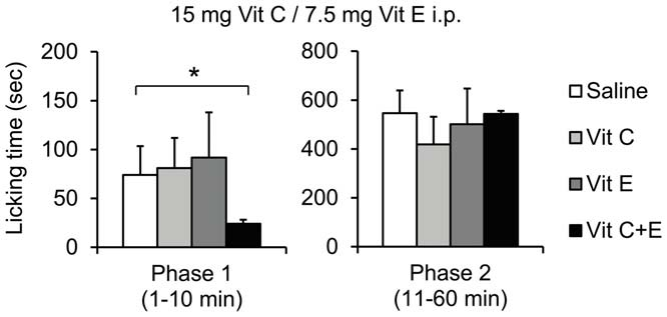
Antinociceptive effects of a Vit C and Vit E combination in the formalin test. Drugs (15 mg Vit C, 7.5 mg Vit E, the combination of 15 mg Vit C and 7.5 mg Vit E, or saline) were intraperitoneally administered 20 min prior to injection of formalin (15 µl, 5%) into a hindpaw. Sum of paw-licking time in phase 1 (1–10 min) and phase 2 (11–60 min). Note that the licking behavior in phase 1 is considerably reduced in mice treated with the Vit C+E combination. *n* = 6–8 per group; *significantly different from saline group, *p*<0.05.

We first assessed whether i.p. injection of Vit C, Vit E or a combination of both vitamins (Vit C+E) affects the nociceptive behavior of mice in three well-characterized models of persistent pain, i.e. the formalin test [18], [19], the Complete Freund's Adjuvant (CFA)-induced inflammatory hypersensitivity [24] and the spared nerve injury (SNI)-induced neuropathic hypersensitivity [25]. Based on studies in rats [27]–[29] we used doses of 15 mg Vit C (corresponding to 0.6 g/kg) and 7.5 mg Vit E (corresponding to 0.3 g/kg) for initial screening. In the formalin test, Vit C, Vit E, Vit C+E or saline were i.p. administered 20 min prior to 5% formalin injection into a hindpaw, and the nociceptive behavior was observed over 60 min. The formalin injection evoked the typical biphasic paw licking behavior. Interestingly, pretreatment with the Vit C+E combination, but not with Vit C or Vit E alone, significantly inhibited the first phase of paw licking (1–10 min), which results from peripheral activation of primary afferents (Fig. 1). In contrast, the second phase of paw licking (11–60 min) that involves a period of sensitization was not affected by the vitamin combination, nor by Vit C or Vit E given alone (Fig. 1). To exclude the possibility that the selective inhibition of phase 1 but not of phase 2 is due to a short duration of action of the vitamins, we also tested the effect of the Vit C+E combination when i.p. administered 2 min prior to formalin injection. Similarly, pretreatment with Vit C+E 2 min prior to formalin selectively inhibited the first but not the second phase of paw licking (data not shown). These data indicate that the combination of vitamins C and E may inhibit formalin-induced peripheral pain processing.

### Vitamins C and E treatment does not inhibit CFA-induced pain behavior

**Figure 2:**
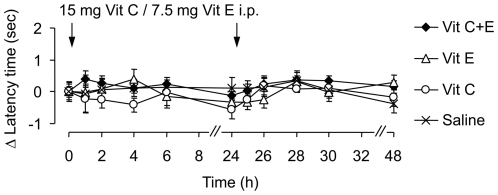
CFA-induced inflammatory pain behavior is not affected by Vit C and Vit E treatment. Mice were injected with 20 µl CFA into a hindpaw. Drugs (15 mg Vit C, 7.5 mg Vit E, the combination of 15 mg Vit C and 7.5 mg Vit E, or saline) were i.p. administered 24 h (time point ‘0’) and 48 h (time point ‘24’) after CFA injection. Paw withdrawal latency times upon mechanical stimulation are expressed as difference to baseline (i.e. prior to the first drug injection). Statistical analyses revealed no significant differences between groups. *n* = 7–8 per group.

We then tested the effect of Vit C and/or Vit E treatment in the Complete Freund's Adjuvant (CFA) model of inflammatory pain. As expected, injection of CFA into a hindpaw evoked mechanical hyperalgesia, indicated by a drop of paw withdrawal latency times from 7.8±0.2 sec before CFA injection to 5.0±0.1 sec 24 h after CFA injection (*P*<0.001). Two doses of Vit C (15 mg), Vit E (7.5 mg), Vit C+E (15 mg + 7.5 mg) or saline were i.p. administered, the first dose 24 h and the second dose 48 h after CFA injection. As shown in Fig. 2, the CFA-induced mechanical hyperalgesia was not affected by Vit C, Vit E or Vit C+E. In contrast, the hyperalgesia was significantly reduced after administration of diclofenac (10 mg/kg i.p.) that was used as a positive control (data not shown). Hence, CFA-induced inflammatory hyperalgesia, in contrast to formalin-induced paw licking, is obviously not inhibited by Vit C+E treatment in the doses given.

### A combination of vitamins C and E attenuates SNI-induced neuropathic pain behavior

**Figure 3:**
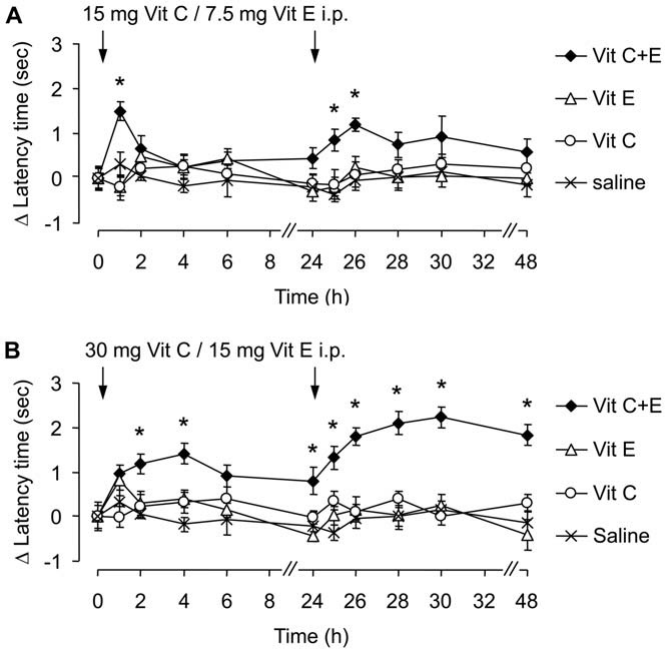
A combination of Vit C and Vit E inhibits SNI-induced neuropathic pain behavior. Mice were subjected to SNI surgery and drugs (Vit C, Vit E, the combination of Vit C and Vit E, or saline) were i.p. administered at the indicated doses 14 days (time point ‘0’) and 15 days (time point ‘24’) after SNI surgery. Paw withdrawal latency times upon mechanical stimulation are expressed as difference to baseline (i.e. prior to the first drug injection). Note that the Vit C+E combination dose-dependently inhibited SNI-induced mechanical allodynia, and that a sustained effect occurred after a second injection of Vit C+E. *n* = 8 per group; *significantly different from saline group, *p*<0.05.

We then analyzed the effect of Vit C and/or E treatment in the SNI model of neuropathic pain. Fourteen days after SNI surgery, paw withdrawal latency times in the SNI-operated hindpaw were 4.4±0.1 sec as compared to 8.3±0.3 sec before SNI surgery (*P*<0.001), indicating mechanical allodynia. Interestingly, i.p. administration of Vit C+E (15 mg + 7.5 mg, respectively) significantly alleviated the mechanical allodynia 1 h after drug injection (Fig. 3A). Moreover, a sustained effect was observed when a second injection of Vit C+E was given 24 h after the first injection. By contrast, administration of Vit C or Vit E alone did not affect mechanical allodynia (Fig. 3A). These data indicate that coadministration of vitamins C and E may inhibit SNI-induced neuropathic pain behavior.

In order to investigate whether the antinociceptive effect of Vit C+E is dose-dependent, we administered twofold higher doses of the vitamins (i.e., 30 mg Vit C and/or 15 mg Vit E) 14 and 15 d after SNI surgery. As shown in Fig. 3B, higher doses of the vitamin combination alleviated the neuropathic pain behavior more effectively than lower doses (compare to Fig. 3A), and stronger effects were again observed after the second drug injection which was given 24 h after the first injection. By contrast, administration of 30 mg Vit C or 15 mg Vit E alone did not affect mechanical hypersensitivity (Fig. 3B). Thus, SNI-induced neuropathic pain behavior was dose-dependently inhibited by systemic administration of a combination of vitamins C and E, but not by administration of Vit C or Vit E alone.

### Intrathecal delivery of vitamins C and E in combination attenuates SNI-induced neuropathic pain behavior

**Figure 4:**
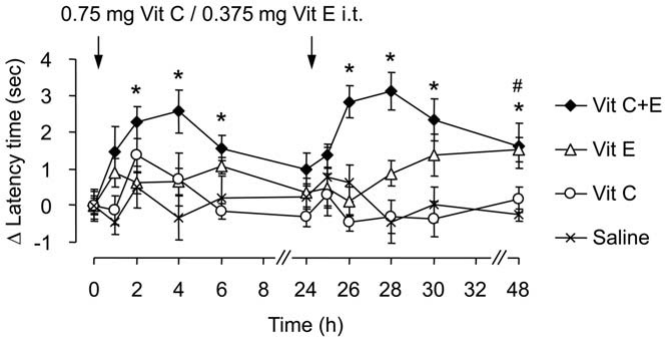
Intrathecally administered Vit C and E inhibit SNI-induced neuropathic pain behavior. Same experimental setting as described in Figure 3, but with intrathecal drug administration at the indicated doses. The Vit C+E combination effectively inhibited SNI-induced mechanical allodynia after i.t. administration. *n* = 6–8 per group; *,^#^significantly different from saline group (*p*<0.05) for Vit C+E and Vit E alone, respectively.

The i.p. administered combination of vitamins C and E may act on multiple sites of the nociceptive system. To determine the importance of the spinal cord as an active site for the attenuation of neuropathic pain behavior, we analyzed the effect of intrathecal (i.t.) administration of Vit C and/or Vit E on SNI-induced mechanical allodynia. Vit C (0.75 mg) and Vit E (0.375 mg) or Vit C+E (0.75 + 0.375 mg), corresponding to 5% of the effective systemic doses, were i.t. injected 14 and 15 days after SNI surgery. Interestingly, the vitamin combination significantly reduced mechanical allodynia over 2–6 h after the first and over 2–24 h after the second i.t. injection, respectively (Fig. 4). Vit E given alone significantly reduced allodynia 24 h after the second i.t. injection, whereas Vit C given alone was without effect (Fig. 4). These data point to the spinal cord as an action site for the antinociceptive effects of the Vit C+E combination during neuropathic pain.

### Vitamins C and E in combination inhibit p38 MAPK phosphorylation in the spinal cord and in DRGs

**Figure 5:**
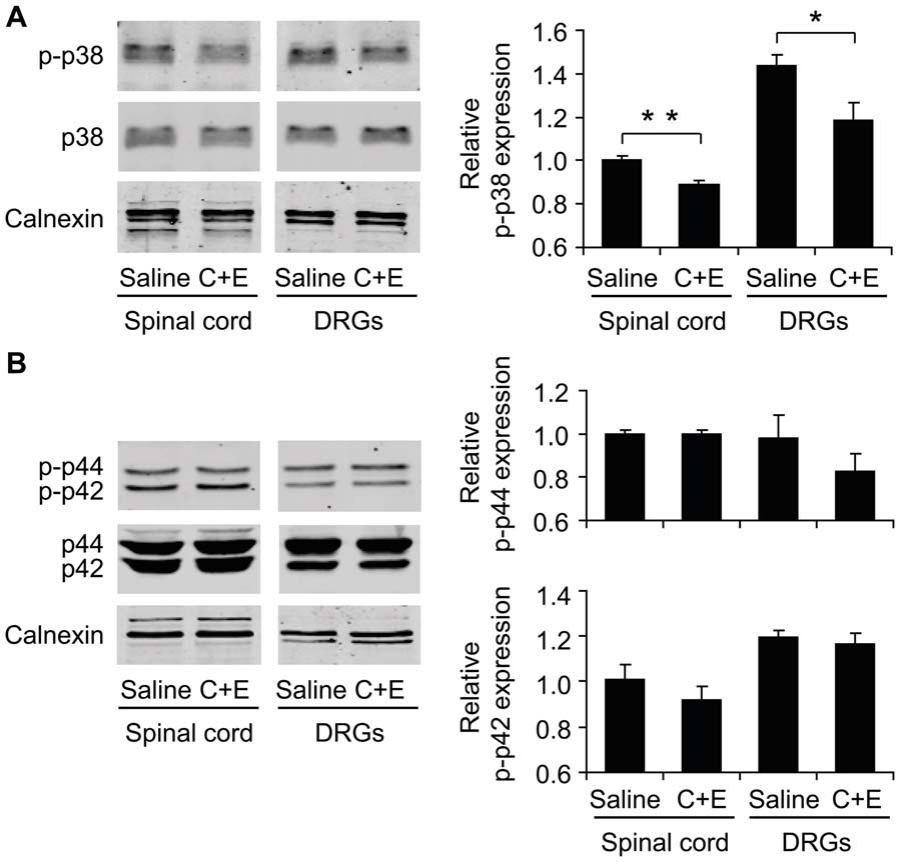
Vit C and Vit E treatment attenuates phosphorylation of p38, but not of p42 or p44, in the spinal cord and DRGs. Mice were subjected to SNI surgery and received two i.p. injections of saline or the combination of Vit C (30 mg) and Vit E (15 mg) at days 14 and 15 after SNI. The protein expression of phospho-p38 (p-p38) and p-38 (A), and of phospho-p42 (p-p42), p42, phospho-p44 (p-p44) and p44 (B) in the spinal cord and DRGs was analyzed by western blotting of tissues obtained 3 h after the second drug injection. Calnexin was used as loading control. Representative western blots are shown on the left, densitometric analyses are shown on the right. *n* = 3 animals per group; *p<0.05, **p<0.01.

Because MAP kinase activation is an important contributor to central sensitization during nociceptive processing [30] and MAP kinases have been suggested as a downstream signaling mechanism of ROS in other tissues [31], we investigated the effect of Vit C+E treatment on p38 and ERK (p42 and p44) MAPK activation in the spinal cord and DRGs by western blot analyses. Fourteen and 15 days after SNI surgery mice were i.p. injected with the Vit C+E combination or saline, and tissues were excised 3 h after the second drug injection, i.e. at a time point of antinociceptive effects of Vit C+E (compare to Fig. 3B). Interestingly, in Vit C+E treated mice we observed significant decreased phospho-p38 protein levels in the spinal cord and in DRGs (*P* = 0.002 and 0.022, respectively) as compared to saline treated mice (Fig. 5A). Furthermore, a trend of decreased p44 phosphorylation was detected in DRGs of Vit C+E treated mice (Fig. 5B), which was however not significant (*P* = 0.137), whereas p42 phosphorylation was not affected. Total p38, p42 or p44 protein in the spinal cord and DRGs did not change after Vit C+E treatment. These data suggest that the antinociceptive effects of Vit C+E on SNI-induced neuropathic pain behavior might involve an inhibition of p38 activation in the spinal cord and in DRGs.

### Allodynia induced by intrathecal administration of a ROS donor is attenuated by vitamins C and E in combination

**Figure 6:**
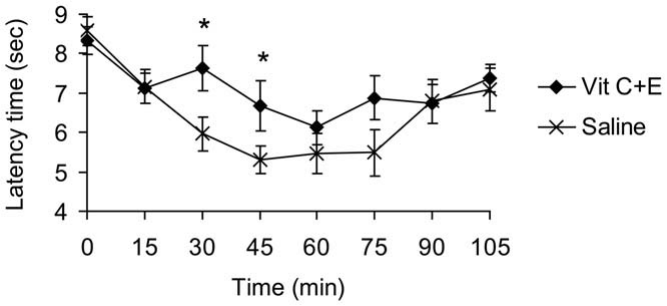
Vit C and Vit E pretreatment attenuates mechanical allodynia induced by intrathecal TBHP. Mice were i.p. pretreated with a combination of 30 mg Vit C and 15 mg Vit E or saline. One hour thereafter, the ROS donor TBHP (100 µg) was i.t. injected (time point ‘0’), and paw withdrawal latency times upon mechanical stimulation were measured for 105 min. *n* = 6 per group; *significantly different from saline group, *p*<0.05.

We then assessed whether the Vit C+E combination inhibits mechanical allodynia induced by i.t. delivery of a ROS donor [14]. Naive mice were i.p. pretreated with a combination of 30 mg Vit C and 15 mg Vit E or saline, and the ROS donor TBHP (tert-butyl hydroperoxide; 100 µg) was i.t. injected 60 min thereafter to induce mechanical allodynia. As shown in Fig. 6, i.t. TBHP evoked transient mechanical allodynia in both groups. However, in mice pretreated with Vit C+E, the TBHP-induced allodynia was significantly attenuated as compared to control mice pretreated with saline (Fig. 6). These data suggest that systemic administration of Vit C and E in combination may inhibit ROS-induced central sensitization.

### Multiple daily administrations of low-dose vitamins C and E in combination inhibit SNI-induced neuropathic pain behavior

**Figure 7:**
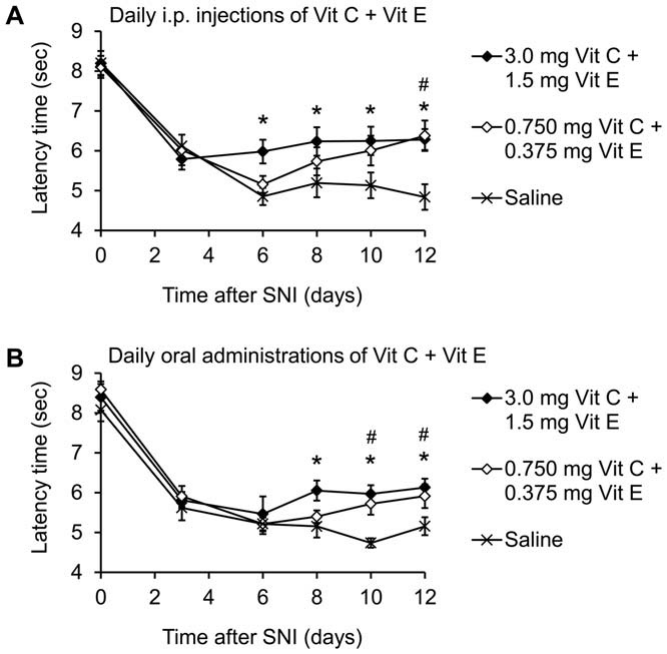
Long-term treatment with low dose Vit C and Vit E inhibits SNI-induced neuropathic pain behavior. Mice were subjected to SNI surgery and drugs were i.p. (A) or orally (B) administered at the indicated doses once daily for 12 days starting immediately after SNI surgery. Paw withdrawal latency times upon mechanical stimulation are shown. Note that both doses of the Vit C+E combination inhibited the neuropathic pain behavior after i.p. and oral administration. *n* = 8 per group; *,^#^significantly different from saline group (*p*<0.05) for the 3 mg + 1.5 mg combination and the 0.75 mg + 0.375 mg combination, respectively.

We next investigated whether Vit C+E at low doses near the human long-term tolerable upper intake level may also reduce the neuropathic pain behavior. For this purpose, mice underwent SNI surgery and were i.p. treated with a combination of low dose Vit C+E or with saline 30 min after SNI surgery, followed by once-daily i.p. administration for 12 days. Interestingly, a combination of 3 mg Vit C + 1.5 mg Vit E significantly inhibited SNI-induced mechanical allodynia during 6–12 days after SNI surgery (Fig. 7A). Moreover, in mice treated with 0.75 mg Vit C + 0.375 mg Vit E mechanical allodynia was gradually attenuated from 8–12 days after SNI surgery with a significant effect after 12 days (Fig. 7A), indicating that antinociceptive effects can be achieved by delivery of low doses of Vit C+E.

We further analyzed whether antinociceptive effects also occur after oral administration of the vitamin combination. Mice were treated by oral gavage with low doses of Vit C+E or with saline 30 min after SNI surgery, followed by once-daily oral administration for 12 days. As shown in Fig. 7B, the SNI-induced mechanical allodynia was also attenuated after oral dosing of the vitamins. Significant effects were observed after administration of 3 mg Vit C + 1.5 mg Vit E, and of 0.75 mg Vit C + 0.375 mg Vit E, respectively (Fig. 7B). Hence, multiple daily i.p. or oral administrations of low doses of Vitamins C and E in combination may inhibit SNI-induced neuropathic pain behavior.

### Multiple daily administrations of low-dose vitamins C and E in combination do not inhibit CFA-induced inflammatory pain behavior

**Figure 8:**
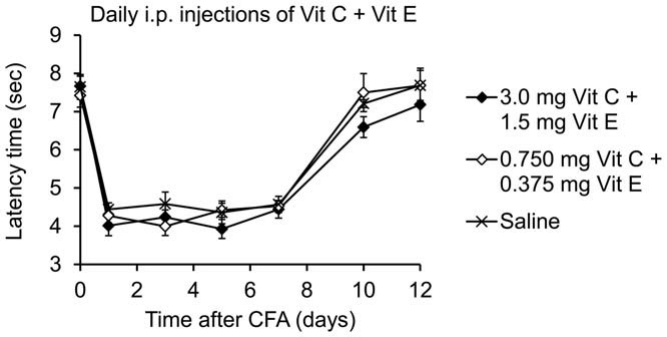
Long-term treatment with low dose Vit C and Vit E does not inhibit CFA-induced inflammatory pain behavior. Mice were injected with 20 µl CFA into a hindpaw and drugs were i.p. administered at the indicated doses once daily for 12 days starting immediately after CFA injection. Paw withdrawal latency times upon mechanical stimulation are shown. Statistical analyses revealed no significant differences between groups. *n* = 8 per group.

Finally, we investigated whether Vit C+E at low doses may reduce the CFA-induced inflammatory pain behavior. CFA was injected into a hindpaw and mice were i.p. treated with a combination of low dose Vit C+E (0.75 mg Vit C + 0.375 mg Vit E; and 3 mg Vit C + 1.5 mg Vit E) or with saline 30 min after CFA injection, followed by once-daily administration for 12 days. The CFA injection evoked transient mechanical hyperalgesia in all groups (Fig. 8). In contrast to the neuropathic pain behavior, the inflammatory pain behavior was not inhibited by daily low-dose Vit C+E treatment (Fig. 8). These data are in parallel with our observation that two injections of high-dose Vit C+E do not affect CFA-induced inflammatory pain behavior (Fig. 2). All together, we conclude that vitamins C and E in combination may inhibit nociceptive processing in special pain states.

## Discussion

In the present study, we show that a combination of vitamins C and E inhibits the nociceptive behavior of mice. Systemically administered Vit C+E, in contrast to Vit C or Vit E alone, attenuated the nociceptive behavior in the formalin test (phase 1) and in the SNI model of neuropathic pain. Systemic Vit C+E also ameliorated the allodynia induced by an intrathecal ROS donor. Antinociceptive effects of intrathecal Vit C+E and a reduced p38 phosphorylation indicate that Vit C+E inhibit neuropathic pain processing in the spinal cord and DRGs.

Antinociceptive effects of Vit E or Vit C have been reported in earlier studies. For example, in the streptozotocin (STZ) model of diabetic neuropathy in rats, very high doses of Vit E (12 g/kg per day as a dietary supplement for three months) ameliorated nerve conduction deficits [32], and Vit E at doses of 0.5–1 g/kg per day for one month improved nerve dysfunction [33]. In a model of alcohol-induced neuropathic pain, Vit E (0.1 g/kg p.o. per day for 10 weeks) inhibited thermal and mechanical hyperalgesia [27]. Kim et al. [29] observed an attenuation of mechanical allodynia after a single i.p. dose of Vit E (0.1–5 g/kg) in the spinal nerve ligation (SNL) model of neuropathic pain in rats. Rosa et al. [34] reported that a single i.p. dose of Vit C (1–10 mg/kg) may inhibit both phases of 2.5% formalin-induced paw licking and the behavioral responses to intrathecal injection of glutamate, NMDA, AMPA, kainate and substance P in mice, indicating that Vit C may produce antinociception by interaction with ionotropic glutamate receptors. Moreover, a recent study revealed that plasma concentrations of Vit C were lower in patients with postherpetic neuralgia than in healthy volunteers and that Vit C treatment decreased spontaneous pain in these patients [35], [36], which is supported by case reports [37], [38]. In contrast to the cited reports, in our study i.p. administration of Vit E or Vit C alone failed to inhibit the pain behavior in all tested animal models. About the reasons for these apparently contradictory findings we can only speculate. One explanation might be that the species and pain models used in the cited studies were different from those used in our study. On the other hand, it is likely that that Vit C and/or Vit E may differentially inhibit some forms of nociceptive sensory processing or that the vitamins inhibit nociceptive processing only in specific pain states. In fact, different efficacies of analgesic drugs across different animal models of pain have often been reported (for example, see Ref. [39], [40]), which further reflects the complexity of nociceptive processing. Nevertheless, because the nociceptive tests in our study have been performed under constant conditions and by the same investigator, our data demonstrate that Vit C and Vit E may act in a synergistic manner. To the best of our knowledge, this is the first demonstration that coadministration of Vit C with Vit E increases the ability of both vitamins to inhibit pain.

Vit E is an antioxidant that is directly involved in scavenging ROS and quenching lipid peroxidation chain reactions that occur during ROS reactions with polyunsaturated fatty acids [41], [42]. Vit E reactions result in the formation of tocopheroxyl radicals that react with other antioxidants to regenerate the active molecule [43]. Although Vit E is located in membranes and Vit C is present in aqueous phases, Vit C works to regenerate Vit E from its radical form, providing an explanation for the synergistic antioxidative effects if vitamins C and E are given in combination. Vit C, in turn, can be regenerated from its radical form via the ascorbate–GSH cycle that uses NADPH generated from the pentose phosphate pathway as a reducing agent [16]. One has to consider that nonantioxidant functions of Vit E have long been reported to contribute to its physiological effects in immune function, cell signaling, regulation of gene expression and other metabolic processes [44]. However, more recent data suggest that most functions of Vit E depend on its antioxidant properties [45]. Our observation that Vit C increases the ability of Vit E to inhibit the nociceptive behavior further points to an antioxidative process as the major mechanism underlying the antinociception mediated by the vitamin combination.

We used relatively high doses of Vit C (15 mg, corresponding to 0.6 g/kg) and Vit E (7.5 mg, corresponding to 0.3 g/kg) in order to screen whether or not Vit C and/or Vit E might affect nociceptive processing. These doses are certainly too high to be clinically used in humans, although PK-PD relationships indicate that differences between efficacious systemic drug exposure levels in rodents and humans can differ by as much as 50-fold [46]. However, a second i.p. injection of Vit C+E 24 h after the first injection considerably increased the ability of Vit C+E to inhibit the neuropathic pain behavior. The most likely reason for this increased efficacy after repetitive administration is a storage of Vit E in adipose tissue, leading to cumulative effects [29], [47]. These observations led us to hypothesize that repeated administration of lower doses of Vit C+E might also exert antinociceptive effects. Indeed, we show that daily i.p. or oral administration of Vit C (0.75 mg, corresponding to 30 mg/kg) and Vit E (0.375 mg, corresponding to 15 mg/kg) attenuate the neuropathic pain behavior. Considering that metabolic rates differ between mice and men, it is tempting to speculate that repeated administration of well-tolerated doses of Vit C+E might also attenuate neuropathic pain in humans, at least in some cases. In adult humans, the tolerable upper intake level (i.e., the highest level of daily nutrient intake that is likely to pose no risk of adverse health effects in almost all individuals) of Vit E is 1 g per day of any form of supplementary alpha-tocopherol, whereas the upper intake level of Vit C is 2 g per day [48]. Notably, intake above the upper intake level may be appropriate for investigation within well-controlled clinical trials employing appropriate safety monitoring of trial subjects [49], [50].

We here provide several lines of evidence that spinal mechanisms contribute to the analgesic effects of Vit C+E. First, the Vit C+E combination was effective in attenuating neuropathic pain after intrathecal injection. Second, systemic administration of Vit C+E attenuated mechanical allodynia induced by intrathecal TBHP delivery. The reversible nature of the allodynia evoked by this ROS donor further demonstrates that ROS may exert specific signaling functions during spinal pain processing [14]. Finally, systemic administration of Vit C+E reduced phosphorylation of p38 MAPK in the spinal cord and in DRGs. There is considerable evidence that the three major members of the MAPK family (p38, ERK and JNK) play a major role in the maintenance of persistent pain [30]. In particular, p38, ERK and JNK are differentially activated in glial cells after peripheral nerve injury, leading to the synthesis of proinflammatory/pronociceptive mediators, thereby enhancing and prolonging neuropathic pain. Phospho-p38 begins to increase in spinal cord microglia at 12 h after nerve injury, reaches a peak at 3 days, but is maintained at elevated levels even after 3 weeks [30]. In DRGs, p38 activation occurs predominantly in neurons [51]. ERK is sequentially activated in microglia (peak between 1 and 3 days post-surgery) and then in astrocytes (peak 21 days post-surgery) of the dorsal horn, as well as in DRG satellite cells [52]. Unlike the activation patterns of p38 and ERK, JNK phosphorylation was primarily observed in spinal astrocytes [53]. Our observation that Vit C+E treatment reduces p38 but not ERK (p42/p44) phosphorylation in tissue extracts from the spinal cord and DRGs of SNI-treated animals indicates that p38-dependent nociceptive signaling in spinal cord microglia and DRG neurons might be inhibited by the vitamin combination. Interestingly, a selective activation of p38 by ROS has been demonstrated in perfused rat hearts [54], and there is evidence that apoptosis signal-regulating kinase 1 (Ask1), a redox sensitive MAP3K that activates p38, mediates this effect [55]. However, the mechanisms by which ROS activate p38 signaling pathways in nociceptive processing remain to be elucidated.

We also demonstrate that systemic Vit C+E delivery inhibits the first phase of formalin-induced paw licking. There is much evidence that this phase is mainly driven by peripheral mechanisms. Recent studies revealed that formalin at low concentration (≤ 0.5%) activates TRPA1, as indicated by a considerably reduced nociceptive response in TRPA1^−/−^ mice [56], [57]. In contrast, the higher concentration (5%) of formalin that is often used in the formalin test of pain behavior, as we did in our study, can recruit both TRPA1- and non-TRPA1-expressing afferents [58]. Interestingly, several key players of nociceptive transduction have been shown to be redox-modulated. For example, the capsaicin receptor TRPV1 is potently activated by endogenous oxidized linoleic acid products, thereby contributing to inflammatory hyperalgesia [59]. Moreover, redox-based posttranslational modifications may also alter the function of TRPA1 channels [60]. It remains to be determined which targets mediate the antinociceptive effects of Vit C+E during the early formalin response.

In contrast to the neuropathic pain behavior and the first phase of formalin-induced paw licking, vitamins C and E failed to affect the second phase of formalin-induced paw licking and the CFA-induced inflammatory pain behavior. This finding is somehow surprising, considering that other studies reported an inhibition of inflammatory pain behavior after administration of free radical scavengers or superoxide dismutase mimetics such as phenyl-N-tert-butylnitrone (PBN), 4-hydroxy-2,2,6,6-tetramethylpiperidine-1-oxy (TEMPOL), N-acetyl-L-cysteine or M40403 [11]–[14]. Moreover, the NMDA receptor, which essentially contributes to nociceptive processing during both inflammatory and neuropathic pain, is redox regulated with at least seven cysteine residues involved [61], and its activity can be inhibited by Vit C administration [34]. These findings implicate that various ROS effectors exist which are specifically targeted by ROS during the processing of inflammatory and/or neuropathic pain, and that different antioxidants may act in a substrate-specific manner. Moreover, different ROS generators might specifically contribute to pain processing. Interestingly, recent reports indicate that members of the NADPH oxidase family, which enzymatically produce superoxide from oxygen using NADPH as an electron donor, are expressed at distinct sites of the nociceptive system. For example, the catalytic NADPH oxidase subunit Nox1 is localized to DRG neurons, whereas Nox2 is expressed in spinal microglia. Notably, both subunits have been reported to contribute to nociceptive processing during persistent pain [8], [9]. In other tissues it has been shown that enzymes including xanthine oxidase and nitric oxide synthases when “uncoupled” by hypoxia or loss or oxidation of their cofactor, tetrahydrobiopterin, may also specifically produce ROS [62]. The relative contributions of different ROS generators and effectors will need to be worked out, but ROS are likely to play a prominent role in the sensitization of pain pathways.

In summary, we here demonstrate that neuropathic pain induced by peripheral injury and the acute pain response to formalin can be inhibited by a combination of Vit E and Vit C. Hence, supplementation or treatment with both vitamins might be an option in patients suffering from specific pain states.
